# Experiences with an Advance Care Planning Intervention for Children with Life-Limiting Conditions: A Qualitative Study of Families and Clinicians Using the IMplementing Pediatric Advance Care Planning Toolkit †

**DOI:** 10.3390/children13040486

**Published:** 2026-03-31

**Authors:** Jurrianne C. Fahner, Johannes J. M. van Delden, Judith C. Rietjens, Agnes van der Heide, Marijke C. Kars

**Affiliations:** 1 Julius Center for Health Sciences and Primary Care, University Medical Center Utrecht, 3584 CX Utrecht, The Netherlands; 2 Department of Public Health, Erasmus Medical Center, 3015 GD Rotterdam, The Netherlands

**Keywords:** advance care planning, palliative care, goals of care

## Abstract

**Highlights:**

**What are the main findings?**

- Clinicians and families value an open exploration of values, goals and preferences regarding the future care and treatment of a child living with a life-limiting condition by using a structured conversation guide.
- Families and clinicians experienced a shared understanding of goals of care after the conversation to a limited extent.

**What are the implications of the main findings?**

- Use of a structured advance care planning (ACP) approach is warranted even in the longer existing patient–clinician relationships.
- Explicitly formulating goals of care and specific next steps by clinicians as outcome of an ACP conversation is essential to support an effective and ongoing ACP process over time.

**Abstract:**

Background: Advance care planning is a strategy to define goals and preferences for future care and treatment aligned to patient values. The IMplementing Pediatric Advance Care Planning Toolkit (IMPACT) provides a holistic, family-oriented approach to involve families of children with life-limiting conditions and their clinicians in ACP, starting early in disease trajectories. This study explores how children with life-limiting conditions, and their parents and clinicians experience ACP conversations based on IMPACT. Methods: A multicenter, qualitative interview study using inductive thematic analysis was conducted. A total of 27 cases of children with life-limiting conditions were included in the study from February 2019 to December 2019. Interviews with 18 clinicians, 24 mothers, 8 fathers and 3 children were conducted. Results: Clinicians and families of children with life-limiting conditions valued to be involved in ACP conversations based on IMPACT. Although it confronted both parents and clinicians with the impact of caring for a child with a life-limiting condition, sharing the family’s narrative resulted in a stronger relation between families and clinicians. This relation was experienced as a good foundation to share values and preferences for future care and treatment. However, a shared understanding of goals of future care, and treatment based on the conversation was experienced to a limited extent. Conclusions: ACP conversations based on IMPACT facilitated family-centered conversations, and were valued by families of children with life-limiting conditions and their clinicians. The meaning of the family’s narrative in relation to goals and preferences for future care and treatment needs ongoing conversations and coaching on the job of clinicians initiating those conversations.

## 1. Introduction

Children with life-limiting conditions and their families balance between a focus on disease and symptom control, and a focus on creating a life worth living for the child and family [1]. Anticipating the future when navigating serious illness is complicated due to prognostic uncertainty, a focus on the here-and-now, and hesitance among clinicians and families to initiate complex conversations about quality of life and end of life [2,3].

Advance care planning (ACP) is emphasized in guidelines and daily practice as a strategy to identify, share, document and review values, goals and preferences for future care and treatment [4,5,6]. In pediatrics, ACP is often promoted as a valuable strategy to define goals of care and align care and treatment to family values; yet ACP is perceived as emotionally challenging. ACP is often postponed to the very end of life [7,8]. This limits the exploration of families’ perspectives on future care and treatment earlier in disease trajectories. Initiation of ACP early in disease trajectories creates opportunities to think about, and share values, goals and preferences without the actual need of immediate decision-making during those early and ongoing conversations [9].

Few well-investigated pediatric ACP interventions exist [10,11]. These focus mostly on the end-of-life themes in specific disease populations, such as oncology patients, children admitted to the intensive care unit and children living with acquired immune deficiency syndrome [12,13]. Some existing interventions are adapted to other specific populations [14,15].

To facilitate ACP in pediatrics for children with life-limiting conditions in general, starting early in disease trajectories and continuing until the end of life, the IMplementing Pediatric Advance Care Planning Toolkit (IMPACT) was developed [16,17]. The development consisted of a five-step process in close collaboration with clinicians and families and entailed a theoretical background [12]. The perspectives of the stakeholders on the structure, content and wording of the IMPACT toolkit were evaluated and adapted where appropriate [16]. IMPACT aims to support clinicians and families to participate in ACP from a holistic point of view, with attendance to the voice of the child and with a caring attitude to families that acknowledges their challenging context during the ACP process. The need for a holistic mindset in pediatric ACP is increasingly acknowledged [9]. IMPACT focuses on what children with life-limiting conditions, and their parents consider important to themselves and as a family when facing their future. This approach involves the physical, psychological, social and spiritual domain. IMPACT intends to support ACP as an individualized, family-oriented process that can overcome clinicians’ barriers to ACP, such as perceived parental unreadiness, fears of triggering intense emotions and taking away hope. IMPACT aims to structure ACP conversations by providing topics and verbal examples to stay close to the families’ perspectives, whereas at the same time it provides clinicians with opportunities to share their expertise regarding the anticipation of the child’s future. Staying close to the families’ perspectives and needs is experienced as challenging by clinicians in complex conversations [18].

Research on ACP in pediatrics is often based on the experiences of clinicians and bereaved parents with ACP in general, without a focus on what the ACP process itself entailed in detail [19,20,21,22]. This limit insights into the perspectives of families and clinicians on specific elements of the ACP process, as they experienced it themselves in actual conversations. Beside this, bereaved parents may look back on their ACP process with a different perspective after experiencing the actual loss of their child. Therefore, we conducted a qualitative study among children with life-limiting conditions, their parents and their clinicians, who were involved together in ACP conversations based on IMPACT, both early and later on in disease trajectories. These experiences with actual conversations based on IMPACT were not published prior and contribute to the understanding of the meaning of actual ACP conversations based on IMPACT for the child’s care and treatment, as perceived by the families and their clinicians, and their perceived usefulness of a standardized ACP approach.

## 2. Materials and Methods

### 2.1. Design

A multicenter, interpretative qualitative interview study using an inductive thematic analysis was conducted to explore early experiences with IMPACT [23,24,25]. The COmprehensive consolidated criteria for REporting Qualitative research (COREQ) were used to structure the study report [26].

### 2.2. IMPACT Intervention

IMPACT provides materials to support the identification, discussion and documentation of values, goals and preferences for care and treatment, starting from the families’ perspective on living with illness. The key element of the intervention is a conversation guide to structure ACP conversations. It supports clinicians to explore the child’s and parents’ perspectives on the child’s identity, living with illness, living a good life, expectations for the future, and preferences for future care and treatment. Besides guidance in exploring the families’ perspectives, it provides prompts to integrate the clinicians’ expertise in the conversation. IMPACT also includes information leaflets about ACP for clinicians, children and families and a two-day clinician training in communication attitudes and skills relevant in ACP.

### 2.3. Study Population

Clinicians caring for children with life-limiting conditions were purposively recruited from five academic pediatric hospitals, the Dutch pediatric oncology center, a pediatric hospice and a pediatric home care organization. Clinicians were eligible for the study participation if they were caring for children with life-limiting conditions as a physician or a specialized nurse, willing to participate in the two-day IMPACT training, and able to perform ACP conversations in their daily practice as part of the study. Life-limiting conditions were defined as conditions where there are no curative treatment options left, and conditions where a cure might be possible, but could still lead to a premature death [27]. Variation was sought with respect to the clinicians’ specialty and experience. After attending the IMPACT training, the clinicians invited the parents of children with life-limiting conditions for an ACP conversation, as considered appropriate by the clinician. The clinicians informed the parents about the study and asked for consent to share their contact information with the research team. One researcher (JF) contacted the families, provided more information about the study and asked for consent to participate. The researcher reported the consent to the clinician, and the clinician planned an ACP conversation as part of the study. Families were eligible for participation if having a child diagnosed with a life-limiting condition, Dutch-speaking and willing to have an ACP conversation about their child. Children themselves participated in the ACP conversation and study, as was indicated as appropriate by the parents and agreed on by the child. In accordance with the Dutch legislations, informed consent was obtained from children of 12 years old and above, if not cognitively impaired. For children under the age of 12 years, informed consent was obtained from the parents.

### 2.4. Data Collection

Data were collected in semi-structured face-to-face interviews with clinicians, parents and children. Data were collected from February 2019 to December 2019. Both clinicians and families completed a background questionnaire. Parents and children were interviewed by JF about their experiences shortly after the ACP conversation. Clinicians were interviewed at the end of the study by JF. The interviews were audio-recorded and transcribed verbatim. Interviews lasted 30–90 min and were conducted at a location of the participants’ preference. An interview guide, based on the literature and expertise of the research team, was used to structure the interviews (See Supplementary File S1). Topics included perspectives on the aim of ACP, perspectives regarding their experiences with the ACP conversations, experiences with the IMPACT materials, and clinician training.

### 2.5. Data Analysis

A thematic analysis was performed to explore the experiences of clinicians, families and children with an ACP conversation based on IMPACT [24]. Researcher triangulation was ensured to improve reliability and validity of the analysis. The thematic analysis consisted of three phases [23,25]. First, the researchers (JF and MCK) individually (re)read the transcripts of five individual interviews to get familiar with common aspects and phrases. Two researchers (JF and MCK) individually analyzed and coded meaningful fragments in light of the research questions and compared interpretations together. The meaning of the separate text fragments was determined by interpreting them in light of the whole interview [28]. Initial codes were recoded, resulting in an adapted code list [24]. During the second phase, new interviews were read and discussed by two researchers (JF and MCK). The code tree was evaluated and adjusted. One researcher (JF) coded all interviews, supported by the software program NVivo (Lumivero, version 12). Lastly, the research team identified key themes and related subthemes. The researchers went back and forth between the different steps to guarantee constant comparison. Code saturation was reached on a conceptual level [29].

## 3. Results

### 3.1. Participants

Eighteen clinicians participated in the IMPACT training. Fourteen of them conducted ACP conversations with a total of 27 families within the study. Twenty-five families participated in the subsequent interviews. The children were involved in the ACP conversations in five cases and in the study interviews in three cases. All 18 clinicians were interviewed at the end of the study. Seven ACP conversations took place at home. Eight conversations were conducted jointly by a nurse and a physician. The children were included at different stages of their disease. The participants’ characteristics are reported in Table 1.

Five main perceptions were identified from the experiences of clinicians and families involved in ACP conversations based on IMPACT. It was observed that: (1) clinicians valued a structured, yet open exploration of the families’ perspectives, (2) parents valued attention to their challenging situation, even though this triggered an emotional response, (3) ACP conversations stimulated a stronger relation between families and clinicians, (4) ACP conversations are experienced as part of an ongoing process throughout the child’s disease trajectories, and (5) an added value of ACP conversations regarding future care and treatment was experienced to a limited extent. Table 2 provides quotations to illustrate the findings.

### 3.2. Clinicians Valued a Structured, Yet Open Exploration of the Families’ Perspectives in ACP

Clinicians mentioned ACP as a helpful approach to explore the perspectives of the child and family regarding their values, goals and preferences of future care and treatment on the physical, psychological, social and spiritual domains. IMPACT helped them to explore these domains in a structured way. This revealed comprehensive insights into the family’s perspectives on living with illness and the meaning of living with illness for the child and the family. Clinicians reported that these deeper insights into the family’s life provided them with a background for better understanding of the families’ preferences for care and treatment. They mentioned discussing the families’ concerns and hopes could help clinicians to support families in future decision-making.

Clinicians experienced their role in the conversation mainly as listeners. They preferred to have the conversation during a stable phase in the child’s disease trajectory, without the need for any decision-making at that moment. That created more space to openly explore the families’ perspectives.

Some clinicians used the IMPACT conversation guide as a hand-held booklet during the conversations. They preferred to follow the structure of the guide closely and they valued having the back-up phrases in front of them in case they would not know what to say during a conversation. Others were reluctant to have the guide visible on the table, being afraid that families would interpret this as a lack of skills. However, clinicians agreed this had more to do with their own perception than with the families’ opinion. Families reported the experience of the use of a guide during the conversation as diligent and accurate.

Four clinicians that participated in the two-day IMPACT training did not have any formal ACP conversations during the study period. However, they reported to have integrated elements of IMPACT into their practice, such as questions about the meaning of living with illness and hopes for the future.

Some clinicians mentioned being involved in ACP confronted them with their own professional role and values. Both physicians and nurses perceived being involved in ACP as an important part of their professional duties. Some nurses mentioned struggling with their role in ACP due to the limited decision-making power. Nurses participating in a pediatric palliative care team felt more confident regarding ACP, since the explorative character of ACP fitted their tasks within the multidisciplinary team. Besides that, they felt that they had more autonomy being involved as case managers. They used the ACP conversations to put forward the families’ perspectives during meetings of the medical team.

### 3.3. Families Valued Attention to Their Challenging Context in ACP, Even Though Emotions Were Triggered

Parents were positive about having the ACP conversation based on IMPACT in general. They felt they were being known and heard during the conversations and valued the attention to their child and family beyond the medical domain. Even when the content of the conversations did not reveal new insights for the parents or clinicians, parents appreciated the time invested to share their narratives in a structured way without constraints of time and without the need for decision-making at that moment. None of the parents experienced the conversation topics as inappropriate or too confrontational. Most parents did not have specific expectations of the conversation in advance. Although they appreciated the information leaflet to prepare themselves for the conversation, and stated the conversation fitted their expectations, most parents had difficulties specifying their expectations. They reflected on the aim of ACP in a generalized manner. Most parents mentioned seeing ACP as a strategy to think about good care for their child.

Most parents mentioned the ACP conversation triggered thoughts about ongoing losses due to the child’s life-limiting condition. During the ACP conversation, parents experienced feelings of loss when talking about prior experiences regarding the child’s care and treatment or when looking back to periods where the child had been in a better condition. This confronted them with beautiful moments that had passed by and with difficult situations they had experienced as a family so far. Confrontation with ongoing losses continued when facing the future during the ACP conversation that yielded perspectives regarding disease progression, maintaining care and the child’s end of life. Many parents mentioned these thoughts had continued after the ACP conversation for a while. As such, participating in ACP was experienced as emotionally intense and energy- taking by most parents, yet they valued the attention to their feelings of ongoing loss as these were perceived as part of their daily life anyway.

Three children participated in the ACP conversations and subsequent interviews. Those children reported they felt to be in the lead during the conversations and valued the attention paid to them as a person and what they considered important for their future. Children did not express any specific emotions in relation to the conversations.

### 3.4. ACP Conversations Stimulate a Stronger Relationship Between Families and Clinicians

Both clinicians and parents mentioned having the ACP conversation had deepened their relationship. Clinicians reported the insight into the families’ perspectives on living with illness helped them to understand the challenging context of the family. This provided them with some insight into the background of the values families shared during the conversations. At the same time, hearing about the families’ dealing with their challenging situation and their expectations for the future gave rise to many emotions. Clinicians perceived the conversations as energy-taking, yet valuable due to the feelings of a stronger connection. They reported this made them feel more satisfied with their job.

For parents, the interest in their personal context and acknowledgement of their challenges in caring for a child with a life-limiting condition, made them more open to sharing their deepest thoughts, including their hopes, fears and worries regarding the future of their child. Some families mentioned that having the conversation at their home showed so much effort of the clinician towards the family, and that this influenced their ability and willingness to share their perspectives in a positive way. Clinicians who did ACP conversations at home were very convinced of the added value of having a conversation at the family’s place, even when taking the substantial time investment into account. They mentioned parents could talk more openly in their own homes and seeing the living environment of the family gave them more insight into their way of living and deepened their connection.

### 3.5. ACP Conversations Are Experienced as Part of an Ongoing Process Throughout the Child’s Disease Trajectories

During the interviews it appeared that clinicians and families did not regard the ACP conversations as distinct events but experienced them as parts of an ongoing process throughout the child’s disease trajectories. Most clinicians and families had known each other for some time. Therefore, some ACP topics had been addressed already along the way, such as perspectives on life-sustaining treatments. However, both parents and clinicians reported to have valued a separate appointment for an ACP conversation, to have been able to discuss topics beyond the medical domain and to have taken a step back from day-to-day issues that need to be discussed during regular consultations. However, in most cases there was no follow-up moment scheduled after the conversation. When clinicians and parents reflected on the ACP conversations during the interviews, some reported experiencing some loose ends after the ACP conversation. This included the need for more clarity regarding goals and preferences for future care, more insight into mutual perspectives regarding specific treatment options and information on a follow-up plan. Clinicians and parents stated they felt free to address these issues during next regular consultations.

### 3.6. Added Value of ACP Conversations Regarding Future Care and Treatment Is Experienced to a Limited Extent

Although both clinicians and families appreciated the ACP conversations, they had more difficulties specifying the added value of the conversations regarding future care and treatment. Some parents mentioned to have valued the explication of preferences for future care and treatment that had previously been shared in a less comprehensive way. Others mentioned the conversation confirmed that their expertise as parents regarding goals of care for their child was seen and respected by the clinician. Some parents mentioned expecting from clinicians to take account of the parents’ preferences for treatment limitations as expressed in the ACP conversation in case their child’s condition might deteriorate. Parents felt they might tend to change their views regarding invasive treatments when facing their child’s death. They feared this might not be in their child’s best interest and expressed the need for someone to keep them focused on their initial wishes. These parents expected clinicians to be honest about the prognosis of their child and to share any new insights regarding their child’s condition with them in relation to their values. Parents expressed the hope their clinician could keep them close to their initial wishes, when the fear of losing their child might drive them towards other perspectives.

Clinicians mainly referred to the gained insights into the families’ perspectives on living with illness as an added value in relation to defining goals and preferences of future care and treatment. Few clinicians said the conversation led to clear goals of care and adequate anticipation of future scenarios. Most clinicians experienced ongoing conversations are needed to achieve a shared understanding of goals of care.

## 4. Discussion

This qualitative interview study assessed the experiences of families of children with life-limiting conditions and their clinicians with ACP conversations based on IMPACT. Families and clinicians reported to have appreciated being involved in ACP conversations structured by IMPACT. This was mainly based on the opportunities the conversations provided to share perspectives of the family on living with illness and what they considered important for their child. Although the ACP conversations confronted parents with ongoing losses, resulting in intense emotions, the conversations were experienced as safe and worthwhile as a moment of reflection on their child’s care and future. Clinicians experienced the perspectives on living with illness as insightful and helpful to understand family preferences and their attitude in decision making. An added value of the conversations regarding future care and treatment was experienced to a limited extent. Parents and clinicians had difficulties reflecting on how the conversation content could inform goals of care and future decision-making.

This study reveals some key lessons about ACP, when using a structured, holistic tool like IMPACT. Whereas research shows that ACP in current practice might still have a disease-oriented approach, including a focus on treatment decisions [30,31], our study shows that providing a structured tool to discuss person-oriented themes, gives valuable insight into the families’ perspectives on their child and family life in the context of living with illness. The exploration of life values could entail a comprehensive foundation for defining goals of future care and decision-making [32]. Parents and clinicians value the family-centered approach in our study and experienced this as an investment in their relationship. It is known that an open patient–clinician relationship with response to both verbal and nonverbal expressions can function as a facilitator in ACP [33,34]. Building on an attitude of caring for each other can be seen as a first step in ACP, which might facilitate ongoing ACP conversations in the future when deterioration of the child might induce the need for complex decision-making [35,36].

The findings of this study, that IMPACT contributes to a family-centered approach of ACP and facilitates a stronger relation between families and clinicians, suggest IMPACT can be used as a tool to start ACP early in disease trajectories [9]. This might be beneficial to further implementation of ACP in pediatrics, since research shows integration of ACP early in disease trajectories is perceived as challenging by clinicians [3]. When conducting more challenging and complex conversations, clinicians tend to rely on their intuition and their own assumptions of a helpful conversation strategy, which can overlook the actual needs of the family in the conversation [16]. A structured approach can provide guidance to clinicians to keep attention to the families’ conversation needs and emotional support.

Although the structure of IMPACT is aimed at defining goals of future care and treatment as key element of ACP [4], this turned out to be difficult to achieve in a single conversation. This is known from the literature, where ACP is increasingly seen as an ongoing process and interventions often entail multiple conversations [37]. However, even when a single conversation mainly includes the exploration and identification of family values, a comprehensive summary might elicit how the content of the conversation could inform next steps in the ACP process, even without defining specific goals of care at that moment. Clinicians should safeguard that shared values and preferences are used to inform future goals of care and decision-making. Clinicians need to clarify the conversation aim for parents and stimulate further thoughts about the future of their child in a way that is aligned to the families’ dynamics [38]. Clinicians sometimes still see treatment decisions as a primary outcome of an ACP conversation [39]. This might limit an open exploration of values, mainly early in disease trajectories, where there might not be an immediate need for certain decisions. A focus on specific treatment decisions may lead to expression of a preference for continuing all therapies, whereas discussing and reporting family values apart from treatment decisions can give a more nuanced insight into goals of care [31,40]. In the model of pediatric ACP, as developed during the development of IMPACT, attention to the expertise of the family on living with illness and living a good life, needs to be integrated with the expertise of the medical team regarding the appropriateness of care and treatment in specific (future) situations to achieve a shared understanding of goals of care, contributing to the quality of care for the child [16]. The IMPACT materials and clinician training focus on the exploration of the families’ narrative to stimulate clinicians to convey an attitude of listening and attention to the perspectives of the family. Clinicians normally tend to have a communication attitude of providing information and directing treatment decisions, with a focus on understanding the illness instead of illness perception [41]. This can hinder an open exploration of the families’ perspective. A recent study showed conversations in advanced illness often contain ‘open door moments’ that can be utilized to proceed with talking about goals of care, when using adequate communication skills [42]. In our study, it was observed that the use of IMPACT stimulated clinicians to focus on the families’ perspective, as intended. However, IMPACT also intends to include the clinician’s expertise into the conversations to achieve a shared understanding of goals of care, as illustrated in the model of pediatric ACP. Although logic models underpinning the mechanisms of the intervention help to understand which behavior leads to favorable outcomes, delivering the intervention remains a highly dynamic and delicate process dependent on personal and interpersonal factors of the child, parents and clinicians [43]. A systematic review on parental preferences for ACP showed that parents value the conversations, yet prefer to stay away from the actual treatment decisions [11]. This focus on family values highlights what is really important to the family, whereas at the same time this might result in conversations with a less clear outcome regarding specific goals of care, as we identified in our study. ACP conversations could contribute to better goal concordant care, when clinicians are able to translate the families’ narrative, including their values, to overarching goals of care and subsequent implications for care and treatment [44,45]. This might be clearer in conversations conducted close to the end of life or in a period of deterioration, compared to conversations early on in a disease trajectory. Recent studies about documentation of ACP show that most notes are made in a period of decline in the child’s condition or close to death [46,47]. Aiming for early integration of ACP, more clarity is needed about clear ACP outcomes that can be documented and reviewed over time. A qualitative analysis of ACP conversations showed a diversity of holistic goals of care expressed by parents [48]. These examples might help clinicians to highlight what is important to parents regarding their child’s care. A survey study showed that clinicians mainly rely on their own intuition when guiding ACP instead of using a standardized approach or structured interventions [49]. This might also contribute to the late initiation of the conversations and less clear outcomes of the conversations apart from treatment decisions close to end of life. Training and coaching on the job might support clinicians in their communications skills to achieve a comprehensive outcome of the conversation [50]. Future research might focus on the use of structured ACP approaches by clinicians in actual conversations and to what level they use the interventions as intended.

This study finds its strengths in a diverse sample of clinicians and families participating, that reflects the usability of the intervention for children with life-limiting conditions in general. Besides this, nearly all families participating in an ACP conversation were willing to participate in the evaluating interview, indicating a high level of engagement regarding ACP research in this study. The findings of the study might be influenced by the selection of participants. Clinicians might have invited families for an ACP conversation and the study that they felt comfortable with, which might have led to more positive experiences during the conversations and a more positive framing of their perspectives on the conversations during the subsequent interviews. This may reduce the external validity of the findings. Besides this it was observed that parents had difficulties recalling the specific content of the ACP conversation. Therefore, their experiences might not reflect experiences with IMPACT alone, but experiences within a longer relationship, in which repeated discussions about future care and treatment took place. Inclusion rate of children in both the conversations and the study interviews was very low. Although the IMPACT intervention provides detailed and specific materials to involve children, this did not result in a high rate of involvement. We did not collect data on the children’s cognitive capacities, nor did we ask for the reasons for non-participation. Although the low participation rate of children might be influenced by the children’s age and capacities, lack of age-appropriate involvement of children needs attention as well as gatekeeping by clinicians and parents [51,52]. Clinicians rated their ability to talk with children lower compared to talking with parents [2]. Training focused on communicative interaction between clinicians and children and a multidisciplinary approach such as involving child life specialists, especially when addressing sensitive topics, might contribute to more awareness regarding involvement of the child, both in research and in practice. A systematic review shows flexibility, keeping close contact and alignment to families’ preferences, might contribute to a higher level of engagement of families in pediatric palliative care research [53]. Some clinicians conducted no or a few ACP conversations within the study. Although they reflected on the use of IMPACT during the interviews, their perspectives might be influenced by a limited use of IMPACT. The design of this study did not include repeated interviews or a longer follow-up of the experiences of clinicians and families. The study did not evaluate clinical outcomes, nor did we analyze information from medical health records regarding documentation of the conversations or provided care over time. It might be interesting to evaluate how the ACP process is continued over time to determine any effects of the intervention on actual care received by the child and family in the end. Future research might entail a case-based approach, following families over time to identify correlations between the ACP conversations and actual care as provided, and clinical outcomes as quality of life, quality of care and quality of end of life. The use of IMPACT in clinical practice over time is currently evaluated in multiple national and international studies.

## 5. Conclusions

Clinicians and parents of children with life-limiting conditions experienced ACP conversations based on IMPACT as a valuable strategy to share the families’ perspectives on living with illness and their values regarding the future of their child. The conversations contribute to a better mutual understanding and bonding as fundament for defining goals of care and decision making. A shared understanding of implications for the actual care of the child could not be specified by the families at this stage, shortly after the conversation. The use of the families’ narrative in defining goals of future care and treatment for children receiving pediatric palliative care might need a more incisive attitude from clinicians during the ACP conversations to achieve a shared understanding of the conversation outcomes and next steps.

## Figures and Tables

**Table 1:** Baseline participants’ characteristics.

	*n* (%) *
Characteristics of health care professionals (*n* = 18)	
Gender Female	18 (100)
Age 40–50 years 50–60 years ≥60 years	12 (67) 3 (17) 3 (17)
Profession Nurse Physician	7 (39) 11 (61)
Working experiences in pediatrics 5–10 years 10–15 years 15–20 years 20–25 years 25–30 years ≥30 years	2 (11) 2 (11) 5 (28) 3 (17) 2 (11) 4 (22)
Subspecialty General practitioner in pediatric hospice Home care Hospice care Intensive care Neurology Oncology Palliative care Profound intellectual and multiple disabilities	1 (6) 2 (11) 1 (6) 3 (17) 2 (11) 1 (6) 3 (17) 1 (6)
Number of conducted ACP conversations in study per clinician 0 1 2 3 4 5 6	4 (22) 3 (17) 5 (28) 4 (22) 1 (6) 0 1 (6)
Characteristics of parents (*n* = 41)	
Parents participating in ACP conversation (*n* = 41) Female	26 (63)
Parents interviewed after ACP conversation (*n* = 32) Female	24 (75)
Age (*n* = 32) ≤29 years 30–40 years 40–50 years ≥50 years	4 (13) 5 (16) 16 (50) 7 (22)
Marital stage (*n* = 41) Married/cohabiting Not cohabiting	38 (93) 3 (73)
Nationality (*n* = 41) Dutch Other	40 (98) 1 (2)
Level of education (*n* = 32) Secondary school Vocational education High school University	10 (31) 8 (25) 10 (31) 4 (13)
Religion (*n* = 40) None Roman Catholic Protestant Islam Jewish Other	14 (35) 7 (18) 6 (15) 4 (10) 1 (3) 1 (3)
Characteristics of children (*n* = 27)	
Gender (*n* = 27) Female	16 (59)
Age at participation in pilot study (*n* = 27) 0–5 years 5–10 years 10–15 years 15–18 years ≥18 years	7 (26) 5 (19) 6 (22) 6 (22) 3 (11)
Diagnosis (*n* = 27) Congenital brain disorder Congenital heart disease Epilepsy syndrome Gastrointestinal disorder Genetic disorder Metabolic disease Neuromuscular disease Oncology Unknown	2 (7) 1 (4) 3 (11) 1 (4) 6 (22) 6 (22) 6 (22) 1 (4) 1 (4)
Child’s age at diagnosis (*n* = 24) <1 year 1–5 years ≥5 years	9 (38) 11 (46) 4 (17)
Siblings (*n* = 27) None 1 2 >2	4 (15) 8 (30) 12 (44) 3 (11)
Children participating in… (*n* = 27) ACP conversation Interview after ACP conversation None of the above	5 (19) 3 (11) 22 (81)

* Percentages may not equal 100 due to rounding.

**Table 2:** Illustrative quotes of main experiences in ACP conversations based on IMPACT.

Theme 1. Clinicians valued a structured, yet open exploration of the families’ perspectives
Specialized palliative care nurse: I focused more on the children themselves. I had already known them for quite a while, but this time we talked more about their future—how they want things to be and what they find important. It was less medical and more personal in approach. I enjoyed that, and I think they did too. Home care nurse: You discuss many things briefly at the beginning and at the end of a regular home care visit, when you’re together for a moment. And now you actually go through everything once, in a structured way. And that also made them think, the mother said later.
Theme 2. Parents valued attention to their challenging situation in ACP conversations, even though emotions were triggered
Case 12 (girl, 13 years, neuromuscular disease): Mother: It was a pleasant conversation. I appreciate it that our clinician knows a bit more about our daughter as a person, who she is apart from her disease and ventilator. You know, you will need each other in difficult times. For me it is important that she (the clinician) knows the bigger picture then. Case 18 (girl, 4 years, metabolic disease): Mother: I deliberately didn’t schedule anything else on the day of the conversation. I know I find these kinds of conversations difficult and that they can bring up a lot of emotions, so I kept my day very calm. That really helped me, because it meant I didn’t have to do anything else afterwards. Case 20 (girl, 1 year, brain tumor): Mother: We’ve been in the hospital a lot and you’ve talked to each other a lot anyway. But I really liked the opportunity to settle down and talk things through, having the time to ask all questions that are on my mind and to think about the future together, without being interrupted.
Theme 3. ACP conversations stimulated a stronger relation between families and clinicians
Pediatric palliative care nurse: During the conversations, there grows a connection, a base for future decision-making. That is much better than meeting in an ad hoc situation. You have time to acknowledge the child as a person and the parents in their parenting roles. I think people appreciate it, so does it feel to me, it stimulates a stronger connection. For me it is a very good way to start a conversation with parents in a structured way. Case 15 (boy, 3 years, neurologic disorder): Mother: I think if these conversations happen more often, if I had a conversation like that in the other hospital before my son was admitted to the intensive care unit, I could have felt some connection with that physician. I could have felt supported, but now she did just turn her back on us.
Theme 4. ACP conversations are experienced as part of an ongoing process
Case 1 (male, 8 years, metabolic disease): Mother: The questions that were asked kept me thinking. About his code status for example. These are issues you take home, some homework to think about. I think it is really important to think about the child’s wishes when he is approaching end of life. Does he have a say? We do not think enough about that.
Theme 5. Added value of the ACP conversation regarding future care and treatment
Case 25 (male, 16 years, gastrointestinal disorder): Father: I can image that we, or maybe every parent, have an instinctive reaction of ‘please, save my child’ when it comes to an emergency situation. But at this moment, when we are not at such a point, I really appreciate that we can think about worst-case scenarios from another point of view. So that our clinician can say to us when the moment is there: “You know we talked about this?” That will help us to remember what we really want for our child.

## Data Availability

The data presented in this study are available only on request from the corresponding author due to privacy and ethical restrictions.

## Supplementary Materials

The following supporting information can be downloaded at https://www.mdpi.com/article/10.3390/children13040486/s1, Supplementary File S1: Semi-structure interview guide.

## Abbreviations

The following abbreviations are used in this manuscript:

ACP	Advance care planning
