# Supplementary material for: Experiences with an Advance Care Planning Intervention for Children with Life-Limiting Conditions: A Qualitative Study of Families and Clinicians Using the IMplementing Pediatric Advance Care Planning Toolkit
Source: Children (Basel). 2026 Mar 31;13(4):486. doi: 10.3390/children13040486 (PMC13114344; doi:10.3390/children13040486)
Supplement: Supplementary file 1 [file children-13-00486-s001.zip › children-4184695-supplementary.pdf]

Supplemental File S1

Experiences with an Advance Care Planning Intervention for Children with Life-Limiting Conditions: A Qualitative Study of Families and Clinicians Using the IMplementing Pediatric Advance Care Planning Toolkit

Jurrianne C. Fahner [1,*], Johannes J. M. van Delden [1], Judith C. Rietjens [2], Agnes van der Heide [2] and Marijke C. Kars [1]

[1] Julius Center for Health Sciences and Primary Care, University Medical Center Utrecht, 3584 CX Utrecht, The Netherlands
[2] Department of Public Health, Erasmus Medical Center, 3015 GD Rotterdam, The Netherlands
* Correspondence: j.c.fahner@umcutrecht.nl

Topic list 1 Pilot phase (parents)

Background child

- Could you tell me something about your child and family? How is you child doing at the moment?

Rating ACP conversation

- How do you value the conversation with your physician/nurse on a scale from 0-10?

Experiences ACP conversation

- What do you think about the conversation?
    - What did you like to talk about? What didn't you like to talk about?
    - Where there any difficult topics to talk about?
    - What topics did you consider most important?
    - Did you miss any topics you consider important?
- Where there any differences between this conversation and the regular appointments with your child's clinician?
- Do you think your child's clinician heard any new information from you during the conversation? Did you hear anything new in the conversation?

- How did you feel during the conversation? Did you feel heard?

Effect ACP conversation

- What do you remember of the conversation?
  - Did you talk about it with others afterwards? With your spouse for example?
  - Did you write anything down after the conversation?
- Did the conversation change your view on your child’s future, disease, treatment or clinician? How do you feel about that?
- Do you think there will follow any changes in your child’s care and treatment based on the conversation? Is there anything you would like to be different based on the conversation?
- Would you like to have a follow-up conversation? What would you talk about then?
- Would you recommend a conversation like this to other children or families?
- How can we improve such conversations?

Materials IMPACT

- What do you think about the information leaflet to prepare for the conversation?
- Did you have any expectations about the conversations in advance?
- Did you use think about the questions in the leaflet prior to the conversation? Did you talk about the questions in the booklet with someone else before the conversation?

Topic list 2 Pilot phase (children)

Background child

- Could you tell me something about who you are? How about your illness at this moment?

Rating ACP conversation

- How do you value the conversation with your physician/nurse on a scale from 0-10?

Experiences ACP conversation

- What do you think about the conversation?
    - What did you like to talk about? What didn't you like to talk about?
    - Where there any difficult topics to talk about?
    - What topics did you consider most important?
    - Did you miss any topics you consider important?
- Where there any differences between this conversation and the regular appointments with your clinician?
- Do you think your clinician or your parents heard any new information from you during the conversation? Did you or your parents hear anything new in the conversation?
- How did you feel during the conversation? Did you feel heard?

Effect ACP conversation

- What do you remember of the conversation?
    - Did you talk about it with others afterwards? With your parents for example?
    - Did you write anything down after the conversation?
- Did the conversation change your view on your future, your disease, your treatment, your family or your clinician? How do you feel about that?
- Do you think there will follow any changes in your care and treatment based on the conversation? Is there anything you would like to be different based on the conversation?
- Would you like to have a follow-up conversation? What would you talk about then?
- Would you recommend a conversation like this to other children or families?
- How can we improve such conversations?

Materials IMPACT

- What do you think about the information leaflet to prepare for the conversation?
- Did you have any expectations about the conversations in advance?

- Did you use the fill-in booklet? Did you talk about the questions in the booklet with someone else before the conversation?

Topic list 3 Pilot phase (clinicians)

Rating ACP conversations

- How do you value the ACP conversations you conducted during the pilot study on a scale from 0-10?

Experiences ACP conversations

- How do you look back on the conversations?
    - What went well?
    - What would you like to do differently?
    - Where there any difficult topics to discuss?
    - To what extent were the conversations different from your regular patient encounters?
    - Did you experience any difficulties to integrate the ACP conversations in your daily practice?
    - How did you feel during the conversations and afterwards?
    - How did your conversation partners react during the conversations?

Effect ACP conversations

- Did the conversations change your view on the child or family?
- What do you get out of the conversations as a clinician?
- To what extent did the conversations influence planning of future care and treatment? Do the conversations have any influence on (medical) decision making?

Materials IMPACT

- What do you think about the IMPACT training? Did you use it in daily practice?
- Did you use the verbal examples during the conversations?
- What do you think about the conversation guide?
- What do you think about the documentation of the conversations?
- Did you use IMPACT (materials or training) in your work apart from the conversations included in the study? Will you use any elements in your work now the study has ended?
- Do you think ACP conversations have to be part of regular care? Why or why not? Who needs to be involved in ACP? What are next steps in the implementation of ACP in pediatrics?
